# Development of a Real-Time PCR for Identification of Brachyspira Species in Human Colonic Biopsies

**DOI:** 10.1371/journal.pone.0052281

**Published:** 2012-12-20

**Authors:** Laurens J. Westerman, Herbert V. Stel, Marguerite E. I. Schipper, Leendert J. Bakker, Eskelina A. Neefjes-Borst, Jan H. M. van den Brande, Edwin C. H. Boel, Kees A. Seldenrijk, Peter D. Siersema, Marc J. M. Bonten, Johannes G. Kusters

**Affiliations:** 1 Department of Medical Microbiology, University Medical Centre Utrecht, Utrecht, The Netherlands; 2 Department of Pathology, Tergooiziekenhuizen, Hilversum, The Netherlands; 3 Department of Pathology, University Medical Centre Utrecht, Utrecht, The Netherlands; 4 Central Laboratory for Bacteriology and Serology, Tergooiziekenhuizen, Hilversum, The Netherlands; 5 Department of Pathology, VU Medical Centre, Amsterdam, The Netherlands; 6 Department of Internal Medicine, Tergooiziekenhuizen, Hilversum, The Netherlands; 7 Department of Pathology, St. Antonius Hospital, Nieuwegein, Nieuwegein, The Netherlands; 8 Department of Gastroenterology and Hepatology, University Medical Centre Utrecht, Utrecht, The Netherlands; Charité-University Medicine Berlin, Germany

## Abstract

**Background:**

Brachyspira species are fastidious anaerobic microorganisms, that infect the colon of various animals. The genus contains both important pathogens of livestock as well as commensals. Two species are known to infect humans: *B. aalborgi* and *B. pilosicoli*. There is some evidence suggesting that the veterinary pathogenic *B. pilosicoli* is a potential zoonotic agent, however, since diagnosis in humans is based on histopathology of colon biopsies, species identification is not routinely performed in human materials.

**Methods:**

The study population comprised 57 patients with microscopic evidence of Brachyspira infection and 26 patients with no histopathological evidence of Brachyspira infection. Concomitant faecal samples were available from three infected patients. Based on publically available 16S rDNA gene sequences of all Brachyspira species, species-specific primer sets were designed. DNA was extracted and tested by real-time PCR and 16S rDNA was sequenced.

**Results:**

Sensitivity and specificity for identification of Brachyspira species in colon biopsies was 100% and 87.7% respectively. Sequencing revealed *B. pilosicoli* in 15.4% of patients, *B. aalborgi* in 76.9% and a third species, tentatively named *“Brachyspira hominis”*, in 26.2%. Ten patients (12.3%) had a double and two (3.1%) a triple infection. The presence of *Brachyspira pilosicoli* was significantly associated with inflammatory changes in the colon-biopsy (p = 0.028).

**Conclusions:**

This newly designed PCR allows for sub-differentiation of Brachyspira species in patient material and thus allows large-scaled surveillance studies to elucidate the pathogenicity of human Brachyspira infections. One-third of affected patients appeared to be infected with a novel species.

## Introduction

Brachyspiraceae are fastidious anaerobic spirochaetes and are the causative agents of intestinal spirochaetosis, a condition that is characterized by the attachment of Brachyspira species to the colonic mucosa.

In animals, *Brachyspira pilosicoli*, *B. hyodysenteriae*, *B. intermedia* and *B. alvinipulli* can cause serious disease[1]–[3], whereas *B. innocens* and *B. murdochii* are considered harmless commensals of the gut-flora. [4] *Brachyspira pilosicoli* infects a wide range of mammals, including humans [5], [6] ^,^ and is the undisputed etiological agent of porcine intestinal spirochaetosis [3], [7], and a well known pathogen causing diarrhoea and dysentery in pigs and chickens.[8]–[10] In humans thus far only two infecting species have been reported in detail: *Brachyspira aalborgi* (which is found exclusively in humans and higher primates) [11], [12] and *B. pilosicoli* (suggesting putative zoonotic potential of *B. pilosicoli*). [8], [13] Additionally, there is limited molecular evidence of a third Brachyspira species that awaits formal confirmation.[14]–[17] Jensen et al. [15] used fluorescent in situ hybridization to identify an uncharacterised Brachyspira species and proposed the name *“Brachyspira christiani”*, but since they did not sequence 16S rDNA, it can not be ascertained that this species is the same as the species describe by Pettersson et al. [16] We therefore propose to refer to the species described in this paper as “*Brachyspira hominis*”, as it has currently only been described in humans.

Symptoms commonly associated with human intestinal spirochaetosis are chronic diarrhoea, abdominal pain, weight loss, faecal mucus and blood-stained faeces but the clinical relevance of these findings has been debated.[18]–[20] Invasive spirochaetaemia has been reported in critically ill and/or immuno-compromised patients. [21], [22].

**Table 1:** Primer sets, length of amplicon, annealing temperature, extension time and PCR-protocol.

Primer set	Name	5′–––3′	Totallength	°C	Time	Purpose
Bspp	Bspp F	TggACTAATACCgCATATACTCTT	82bp	55	20s	Species discrimination
	Bspp R	TAggCCgCAggCTCAT				
PhHV	PhHV F	gggCgAATCACAgATTgAATC	89bp	55	20s	DNA extraction control
	PhHV R	gCggTTCCAAACgTACCAA				
β-globulin	GH20	gAAgAgCCAAggACAggTAC	268bp	62	60s	DNA quality control
	PC04	CAACTTCATCCACgTTCACC				
Step A	StepA F	TggATAAgTTAgCggCgAACTg	212bp	62	60s	First 16S sequencing primer
	StepA R	TCAggTCggCTACCTATCg				
Step B	StepB F	gAgCCTgCggCCTATTAgC	253bp	62	60s	Second 16S sequencing primer
	StepB R	gCCgAggCTTACATTATCTACTgTC				
Step C	StepC F	AgCgACATCgCgTgAgg	258bp	62	60s	Third 16S sequencing primer
	StepC R	TCCATCATCCCCTACAATATCCAAg				
Pilo-A	Pilo F	AgTTTTTTCgCTTCACgATgAg	90bp	62	60s	*B. pilosicoli* 16S amplification
	StepA R	TCAggTCggCTACCTATCg				
A-Pilo	StepA F	TggATAAgTTAgCggCgAACTg	145bp	62	60s	*B. pilosicoli* 16S amplification
	Pilo R	gCTCATCgTgAAgCgAAAAAAC				
Aalb-A	Aalb F	gACgCTAAAgCgTAgTAgAgg	113bp	62	60s	*B. aalborgi* 16S amplification
	StepA R	TCAggTCggCTACCTATCg				
A-Aalb	StepA F	TggATAAgTTAgCggCgAACTg	120bp	62	60s	*B. aalborgi* 16S amplification
	Aalb R	CCTCTACTACgCTTTAgCgTCA				
Homi-A	Homi F	CTCTTgACACATAAgTgTAgTAgAg	118bp	62	60s	*“B. hominis”* 16S amplification
	StepA R	TCAggTCggCTACCTATCg				
A-Homi	StepA F	TggATAAgTTAgCggCgAACTg	120bp	62	60s	*“B. hominis”* 16S amplification
	Homi R	CCTCTACTACACTTATgTgTCAAgAg				

**PCR conditions**

Primer concentration: 2,5 µM of both forward and reverse primers.

Buffer composition: LightCycler® 480 SYBR Green I Master.

**PCR volume**

15 µl buffer and 5 µl DNA-eluate per PCR.

**PCR consumables**

White 96-well plate (Roche Diagnostics, Almere, The Netherlands) sealed with transparent self-adhesive foil (Roche Diagnostics, Almere, The Netherlands).

**PCR protocol**

Pre-incubation at 95°C for 10 minutes, de-annealing at 95°C for 10 seconds, 10 seconds annealing at the above-specified temperature, amplification at 72°C for the above specified time, 45 cycles. Melting-curve analysis: 5 seconds at 95°C, one minute at 65°C, 2.2°C per second increase of temperature until 97°C with five acquisitions per degree Celsius.

The pathogenic potential of these bacteria in humans has never been thoroughly investigated. Due to the strict anaerobic growth requirements and long incubation times it is difficult to routinely culture Brachyspira species. [12], [23] Therefore, routine diagnosis of human intestinal spirochaetosis is currently only based on histopathology of colonic biopsies (figure S1) and the absence of distinct morphological hallmarks prohibit the discrimination between pathogenic and non-pathogenic Brachyspira species. In addition, the invasive nature of colonoscopy limits epidemiological as well as non-invasive clinical follow-up studies. As a result not much is known on the species distribution of Brachyspiraceae in human infections. Based on histopathological examination of colon biopsies obtained for various reasons the estimated prevalence in the general Western population varies from 1.1% to 6.9%, with higher incidences among immunocompromised patients.[24]–[26] Some studies have been performed using species-specific primers, investigating the epidemiology of *B. aalborgi* and *B. pilosicoli* in humans. Based on these, *B. aalborgi* causes human infection more frequently (80%–88.6%) than *B. pilosicoli* (5.7%–14.3%), however, no data exists on infections with *“B. hominis”*.[27]–[29].

**Figure 1:**
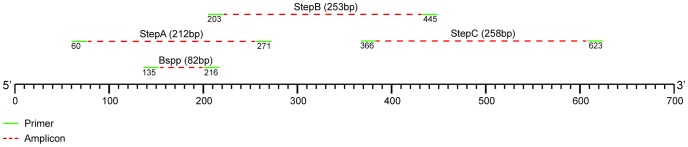
Schematic representation of the relative position of the Brachyspira primers and resultant PCR fragments. Numbering and fragment sizes according to the 16S sequence of the type strain of *B. aalborgi* (NCTC 11492). Fragment names refer to Table 1.

Several PCRs for detecting Brachyspira in faecal or cadaver specimens of animals have been published. [30] These are neither validated for human diagnostic use, nor allow for immediate species determination. To our knowledge, only one PCR has been described for use on human faeces. [31] However, due to the size of the amplified fragment, this PCR is neither suited for use on formalin-fixed paraffin-embedded biopsy samples nor does it allow for real-time species identification. In addition, these PCRs have been species specific, thus only amplifying 16S rDNA from one species and do not allow for the identification of multi-infections with hitherto unknown Brachyspira species.

**Figure 2:**
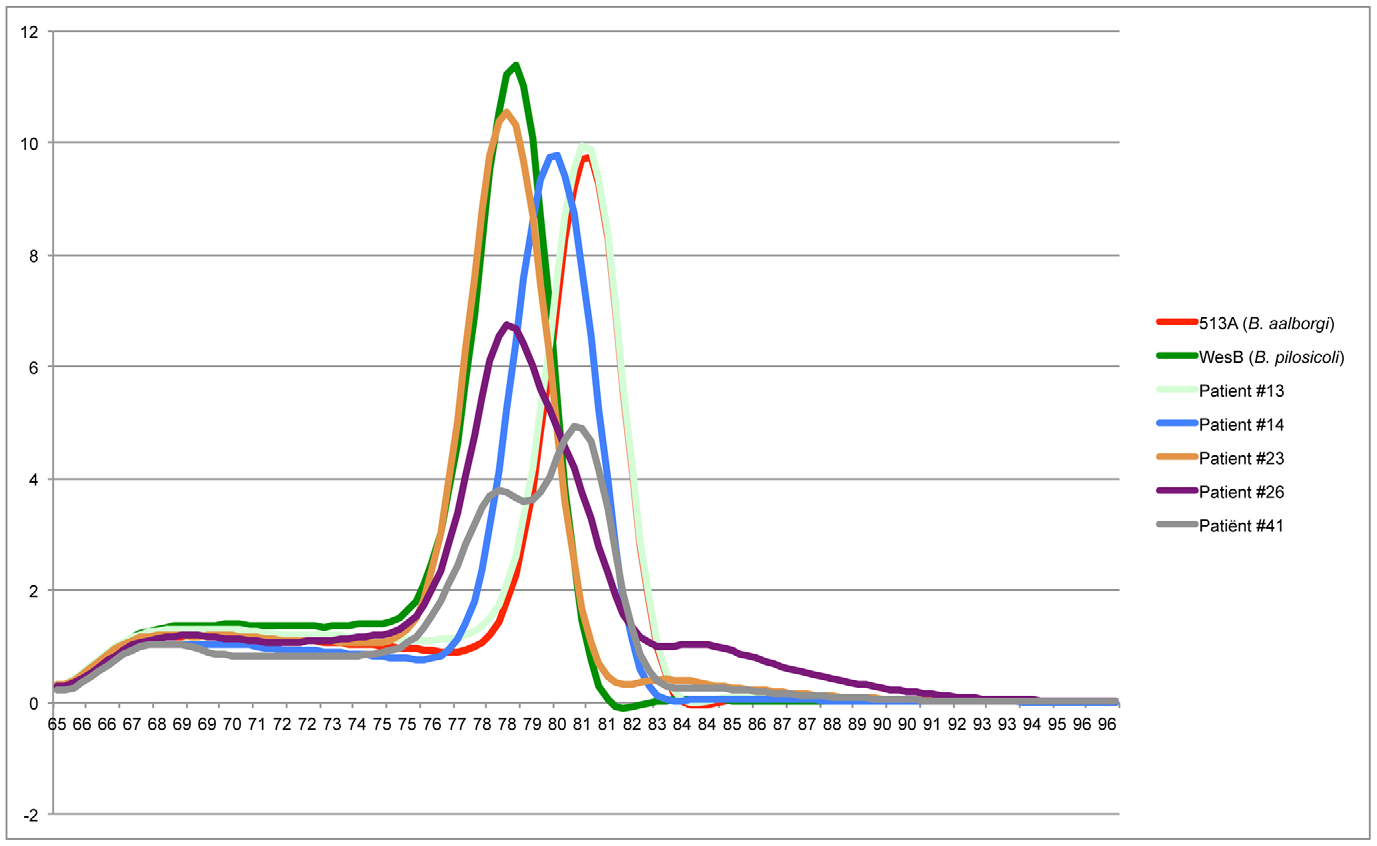
Example of the melting-curve analysis. There is a clear difference in melting-temperature between the type-strain of *B. pilosicoli* and *B. aalborgi*. Biopsy samples containing these species fall neatly within these graphs (patients #13 and #23). *“B. hominis”* (patient #14) is visible as a peak between the type strains, indicating a different base-pair composition. The double infection (patient #26) begins as a *B. pilosicoli*-peak with a clear shoulder under the *“B. hominis”* peak, indicating that two different DNA molecules are present, each with a different melting-temperature-peak. The triple infection (patient #41) is displayed here by the biopsy sample that had both a *B. pilosicoli* and a *B. aalborgi*, another biopsy sample taken from this patient at the same time indicated an infection with *“B. hominis”* (data not shown).

### Aims of this Study

In this study we will describe the design and validation of a real-time PCR allowing species differentiation of Brachyspira species in formalin fixed paraffin tissue specimens and faeces. In addition we investigate species distribution and symptoms in a cohort of patients with histologically documented Brachyspira infection.

**Table 2:** Comparison of melting-curve prediction to sequence result*.

*Melting-curve prediction*	*Sequence result*		
	*B. pilosicoli* (n = 7)	“*B. hominis*” (n = 7)	*B. aalborgi* (n = 41)
*B. pilosicoli* (n = 9)	7	2	0
“*B. hominis*” (n = 11)	0	5	6
*B. aalborgi* (n = 35)	0	0	35

* Multi-infections are not shown in this table, since all multi-infections were predicted correctly.

## Methods

### Ethics Statement

Patients visiting a Dutch hospital are actively informed of the ‘opt-out’ system regarding research on archival patient material. Dutch law requires all studies using such materials to obtain an official approval by the local ethics committee. This study was approved by the Medical Ethics Testing Committee of the University Medical Centre Utrecht, The Netherlands, under protocol number 11-402/C. Only material from patients who did not opt-out has been included in this study.

**Figure 3:**
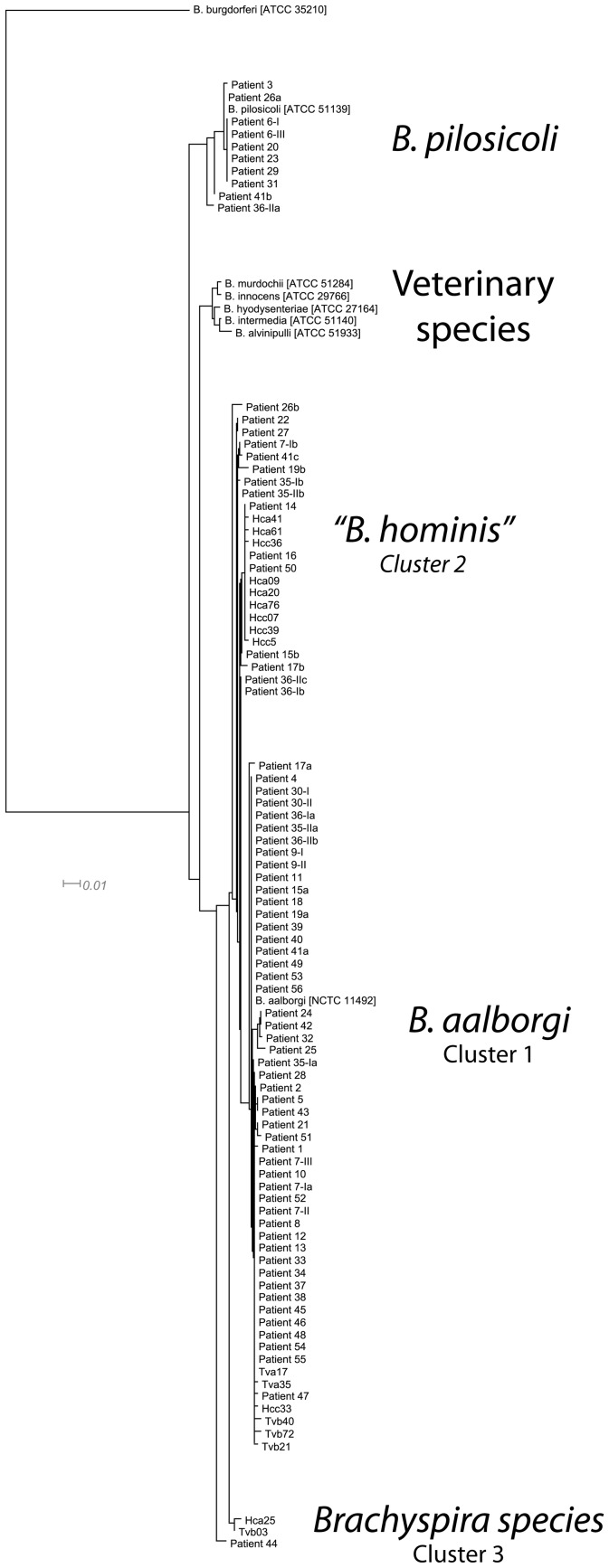
Phylogenetic tree constructed of the first 517 unique base pairs of all patient samples. The sequences published by Pettersson and colleagues (Hcc, Hca Tva and Tvb) and the sequences of the known type strains. The *“B. hominis”* group clearly differs phylogenetically from the known type strains and falls within the group described by Pettersson. Note that two patients (#6-II and #57)) are missing from this tree, since it proved to be impossible to sequence the middle part of the first 517bp of the 16S rDNA. *Borrelia burgdorferi* (ATCC 35210) was used as out-species.

### Sample Selection

Formalin-fixed paraffin-embedded (FFPE) colon tissue taken between 2001 and 2011 was retrospectively identified in four Dutch hospitals (two regional hospitals (Tergooiziekenhuizen, Blaricum/Hilversum and St. Antonius Hospital, Nieuwegein) and two large academic centres (the University Medical Centre, Utrecht and Vrije Universiteit Medical Centre, Amsterdam). Samples were included if the pathology report reported the presence of spirochaetosis and if this was confirmed by immunohistochemistry (*Borrelia burgdorferi* (Lyme) IgG antiserum, ILP 0301, ImmunoLogic, Duiven, The Netherlands). This staining is based on the immunological cross-reactivity of a universal spirochaete antigen and is used routinely in most Dutch laboratories to confirm the diagnosis of spirochaetes in histological samples. As control samples, spirochaete negative colon biopsies, taken between January 2009 and December 2010, were selected if tissue quality was macroscopically good and no spirochaetes were detected by immunohistochemistry.

**Table 3:** Statistical Analyses of Clinical Data^#.^

	*B. pilosicoli* (n = 6)	*B. aalborgi* (n = 38)	*“B. hominis”* (n = 11)	p-value
Diarrhoea	5 (83,3%)	26 (68,4%)	5 (45,5%)	0,230
Abdominal pain	6 (100%)	24 (63,2%)	8 (72,7%)	0,185
Blood loss	4 (66,7%)	13 (34,2%)	3 (27,3%)	0,241
Weight loss	2 (33,3%)	10 (26,3%)	3 (27,3%)	0,938
Mucus	3 (50%)	11 (28,9%)	0 (0%)	0,781
HIV	2 (33,3%)	9 (23,7%)	2 (18,2%)	0,052
Inflammatory changes*	4 (57,1%)	7 (15,6%)	2 (13,3%)	0,028
Two or more complaints	5 (83,3%)	25 (65,8%)	8 (72,7%)	0,660
Three or more complaints	5 (83,3%)	15 (39,5%)	3 (27,3%)	0,071
Four or more complaints	3 (50%)	7 (18,4%)	1 (9,1%)	0,119
Five complaints	0 (0%)	2 (5,3%)	0 (0%)	0,629

# There were 8 patients with double infections (B. pilosicoli+“B. hominis” n = 1; B. aalborgi+“B. hominis” n = 7) and 2 with triple infection. These were analysed in all groups.

Chi-squared test was used in all analyses.

Faecal samples were omitted from analyses.

* Inflammatory changes is calculated for all biopsy samples (*B. pilosicoli* n = 7; *B. aalborg*i n = 45 and “*B. hominis*” n = 15), since this could be established for all biopsy samples.

All colon-biopsy materials were revised by two experienced gastrointestinal pathologists (MEIS and HVS), who were unaware of the initial diagnosis and PCR-results. The presence or absence of inflammatory cells in spirochaete-positive biopsy samples was scored by one pathologist (HVS). Where possible, relevant clinical data were retrieved from clinical files of patients with Brachyspira infection (i.e., sex, age at time of biopsy, indication for biopsy, HIV-status, and presence or absence of rectal blood loss, diarrhoea, abdominal pain, weight loss or faecal mucus).

From three patients, faecal samples were obtained. DNA from these samples was isolated using the same protocol as for the FFPE samples, and further analysis was identical to the colon biopsies.

### Processing of FFPE Biopsy Samples

Of each FFPE-sample, three 4 µm sections were cut for pathological evaluation, and, subsequently, five 20 µm sections were cut and placed in a 1,5 ml Eppendorf tube (Eppendorf, Hamburg, Germany), and three additional control sections were cut (4 µm). The knife was then discarded and the microtome thoroughly cleaned with 96% ethanol to prevent the next FFPE-sample to be contaminated with DNA from the previous sample. The five 20 µm sections were frozen at −80°C until DNA isolation. The six 4 µm sections were fixed on glass slides and archived at room temperature.

### DNA-isolation

DNA-isolation from FFPE samples was performed by standard procedures. These consisted of the removal of the paraffin material through several wash steps followed by automated DNA extraction. The first wash step consisted of adding 1 ml of xylene and fifteen minutes incubation in a shaking heat block (1,400 rpm, 22°C). The samples were then centrifuged at 13,000 *g* for five minutes and subsequently the xylene was removed. This xylene wash-step was performed twice. Then 1 ml 96% ethanol was added to the pellet and samples were vortexed for 5 seconds. Subsequently, samples were centrifuged at 13.000 *g* for five minutes after which the ethanol was discarded. The ethanol wash-step was performed twice. The final wash-step consisted of adding 1 ml acetone, vortexing for 5 seconds, centrifuging at 13.000* g* for 5 minutes and carefully removing the acetone. The pellet was then allowed to dry for 30 seconds at room temperature. Subsequently, 360 µl digestion buffer (10 mM hydroxymethyl-aminomethane, 1 mM ethyleendiamine tetracetic acid, 0.001% sodium dodecyl sulphate and 0.5% Triton X 100) and 20 µl protein K (600 U/ml, Roche Diagnostics, Almere, The Netherlands) was added to the pellet. Finally 40 µl Phocine Herpes Virus (PhHV) DNA was added as an internal control to monitor the DNA isolation and PCR amplification processes. [32] The resultant suspension was placed in a shaking heat block (750 rpm, 70°C) for 30 minutes to allow digestion of tissue and subsequently incubated at 95°C for 15 minutes and 750 rpm to inactivate the protein K. The samples were then stored at −80°C until DNA isolation. DNA was isolated using the MagNA Pure 96 DNA and Viral NA Small Volume Kit on a Roche MagnaPure96 (Roche Diagnostics, Almere, The Netherlands) with the Viral NA Universal SV extraction protocol according to the manufacturer’s instructions.

For the isolation of DNA from faecal samples a suspension of 25 mg faeces in 200 µl lysis buffer (cobas® PCR Female Swab Sample Kit, Roche Diagnostics, Mannheim, Germany) was prepared and spiked with 40 µl PhHV DNA, mixed well and placed at −80°C for more than 2 hours. The frozen solution was then incubated at 95°C for 30 minutes and DNA was extracted using the MagNA Pure 96 DNA and Viral NA Small Volume Kit on a Roche MagnaPure96 (Roche Diagnostics, Almere, The Netherlands), with the Viral NA Universal SV extraction protocol according to the manufacturers instructions.

### Primer-design

Sequences of the small ribosomal subunit DNA of all Brachyspira species and *Staphylococcus aureus* (ATCC 12600; S000529044), *Peptostreptococcus anaerobius* (NCTC 11460; S000140702), *Bacteroides fragilis* (ATCC 25285; S000528922), *Bifidobacterium longum* (ATCC 15697; S000414117), *Escherichia coli* (ATCC 11775T; S000004313), *Pseudomonas aeruginosa* (DSM50071; S000010427), *Borrelia burgdorferi* (ATCC 35210; S000436531) and the 18S of *Candida albicans* (GenBank ID 4512045), *Zae mays* (GenBank ID 6273844) and *Homo sapiens* (GenBank ID M10098.1) were retrieved from the Ribosomal Database Project (release 10, update 22, August 30, 2010) [33] and GenBank (Release 181.0 (14/12/2010)) and aligned using Seaview (version 4.2.8). [34], [35] Four Brachyspira-specific primer-sets were designed based on this alignment (table 1) and analysed in silico for undesirable targets with the Probe Match function of the Ribosomal Database Project and the Primer-BLAST-algorithm. [36] This primer-set was designed to amplify an 82 base-pair region that is specific for both *B. pilosicoli*, *B. aalborgi*, and the putative *“B. hominis”* with the conditions that 1) the primer binding sites are conserved in all Brachyspira species to ensure that all Brachyspira species will be amplified, and 2) the primer binding sites differ sufficiently in sequence from all other known bacterial 16S rDNA sequences so that only 16S from Brachyspira species will be amplified, and 3) the region between these primers differs in length and/or base-pair composition between the three human Brachyspira species thus generating sufficient differences in melting-temperatures of the PCR fragments, allowing for a melting curve based species identification. This was verified with Clone Manager 9 Professional Edition (version 9.0), which uses the expression by Freier et al. [37] Subsequently, this theoretical difference was confirmed using the available human reference strains (*B. pilosicoli* (WesB) and *B. aalborgi* (513A^T^). Since *“B. hominis”* has only been described molecularly no (type or reference) strains where available, and therefore it was not possible to test the correctness of the *“B. hominis”* melting temperature prediction.

Three sets were designed for the generation of Brachyspira specific 16S rDNA fragments thus allowing to obtain the sequences of the first 517bp of 16S rDNA (StepA, StepB and StepC) of the infecting Brachyspira species from the DNA isolated from the biopsies.

Based on results obtained in this study, six primer sets were designed to allow for the generation of Brachyspira species-specific 16S rDNA fragments in double-infected samples for sequencing (Pilo-A, Aalb-A, Homi-A, A-Pilo, A-Aalb and A-Homi). All primer-sets are shown in table 1 and figure 1.

PCR reactions were performed in a LightCycler 480 II PCR machine (Roche Diagnostics, Almere, The Netherlands) according to the manufactures instructions and PCR conditions and primers are summarized in table 1 and figure 1. All primers were synthesized by TIB MOLBIOL GmbH, Berlin, Germany. All samples were tested twice in independent PCRs.

#### Controls and DNA amplification

In addition to the PhHV as a control for the amplification and isolation of DNA, a commonly used and validated β-globulin primer-set was used as control for the successful isolation of DNA from spirochaete-negative FFPE-samples. [38] DNA from the type strains *B. hyodysenteriae* (B-78; ATCC 27164), *B. innocens* (B256; ATCC 29768), *B. aalborgi* (513A; NCTC 11492), *B. pilosicoli* (P43/6/78; ATCC 51139 and WesB), *B. intermedia* (PWS/A; ATCC 51140), *B. murdochii* (56–150; ATCC 51284) and *B. alvinipulli* (C1; ATCC 51933) (kindly donated by dr. D.J. Hampson and dr. T. La, Murdoch University, Perth, Australia) were used as controls. PCR amplification and melting-curve analysis was performed on a Roche LightCycler 480 II (Roche Diagnostics, Almere, The Netherlands) using the protocol supplied in table 1. SYBR-green based melting-curves were generated to assess the T_m_ of the PCR-product were generated with the LightCycler® 480 Software (version 1.5.0.39).

### Sequencing

To allow for the sequencing of the Brachyspira species three primer-sets (Step A, B and C; table 1, figure 1) were designed, that, when assembled, cover the first 517bp of the 16S rDNA. In case of a double infection, species-specific primers (table 1) were used to generate separate PCR products for the individual Brachyspira species predicted to be present in these samples. Sequencing of the PCR products was performed using the BigDye Terminator v1.1 technique on a 3730 DNA Analyser (Applied Biosystems, Carlsbad, California, United States of America) according to the manufactures instructions. Sequences were manually assembled using Chromas (version 2.33, Technelysium Pty Ltd, Australia) and and Seaview (version 4.2.8) and compared with the known sequences available through the Ribosomal Database Project and BLAST-algorithm.

### Construction of Phylogenetic Tree

The assembled sequences and the type strains from all Brachyspira species obtained from the Ribosomal Database were aligned with the aid of Seaview (version 4.2.8) [34], [35], [39] and a phylogenetic tree was constructed from these alignments with SplitsTree (version 4.11.3, built February 26, 2010) [40] using *Borrelia burgdorferi* (ATCC 35210) as an out-species.

## Results

### PCR Validation

The Bspp and StepA-C sets amplified the expected regions in the Brachyspira species type strains (*B. hyodysenteriae* (B-78; ATCC 27164), *B. innocens* (B256; ATCC 29768), *B. aalborgi* (513A; NCTC 11492), *B. pilosicoli* (P43/6/78; ATCC 51139 and WesB), *B. intermedia* (PWS/A; ATCC 51140), *B. murdochii* (56–150; ATCC 51284) and *B. alvinipulli* (C1; ATCC 51933)). When evaluating the melting curves for the Bspp primer-set, there appeared to be a clear difference in melting curve temperature in all three human species (figure 2). The species-specific sets only generated a product in *B. aalborgi* (for Aalb-A and A-Aalb) and *B. pilosicoli* (for Pilo-A and A-Pilo). For the *”B. hominis”*-specific set, this could not be tested in vitro, because to our knowledge no strains of *“B. hominis”* are available.

### PCR Results

Based on the inclusion criteria 63 FFPE-samples from 57 patients with Brachyspira infection and 28 control samples (from 28 patients) were retrieved. Patients with and without Brachyspira infection had comparable age (46.2±14.9 years and 47.4±15.8 for controls; p = 0.716) and sex distribution (p = 0.506). Thirteen infected patients and one control had HIV (p = 0.009). Three biopsies, from one infected and two controls, were excluded from analysis because of negative internal control reactions. For both FFPE and faecal samples, PCR data matched with histopathological results.

### Sequencing, Melting-curve Analysis and Phylogenetic Analysis

The Bspp primer-set produced a product in all samples from histopathologically positive patients and no product was produced in negative controls. Interpretation of the melting-curves is shown in table 2, compared to sequence results. With the exception of two samples (patient 6-II and patients 57), all PCR-positive samples were successfully amplified using primer-sets StepA-C, thus allowing the assembly of the first 517bp of the 16S rDNA. Of patients 6-II and 57, the central part (StepB) of the sequence could not be amplified despite repeated attempts. However, the first (5′-)part could be obtained (StepA) and this contains the most species-specific information, allowing for proper species identification. Based on the sequence results, *B. aalborgi* was identified in 50 (76.9%), *B. pilosicoli* in 10 (15.4%) and *“B. hominis”* in 17 (26.2%) samples. There were eight (12.3%) double infections and two (3.1%) triple infections (table 2). The obtained sequences were submitted to GenBank (accession numbers JX446497 through JX446571, table S1). Using the complete 517bp 16S rDNA sequences a phylogenetic tree (figure 3) was constructed, with the addition of the sequences described by Pettersson and colleagues (named Hca, Hcc, Tva and Tvb followed by a number). [16] In this tree, two lineages consisting of novel Brachyspira sequences can be indicated (as per Pettersson et al.: cluster 1 (*B. aalborgi*), cluster 2 (*“B. hominis”*) and cluster 3 (Brachyspira species). Patient 44 differs from the type strain of *B. aalborgi* by 14 base-pairs, and is closest related to Tvb03, with a difference of nine base-pairs.

When comparing sequence results to melting-curves, melting curves correctly identified the species present in 57 of 65 FFPE samples. In eight samples a double infection and in two samples a triple infection was predicted and in all cases confirmed by sequence analysis. No discrepancies were observed between the duplicate PCRs of the samples. Misclassification occurred in eight samples; six predicted *“B. hominis”* appeared *B. aalborgi* on sequence analysis and two predicted *B. pilosicoli*’s appeared *“B. hominis”* (Table 2).

### Correlation between the Infecting Species and Clinical Symptoms

Clinical information was available from 49 patients with Brachyspira infection (table 3). No significant associations were found except that inflammatory changes were more frequently observed in the presence of *B. pilosicoli* (p = 0.028; Chi-squared test).

## Discussion

We have designed a real-time PCR for species-specific identification of Brachyspira species in formalin-fixed paraffin-embedded material and faeces, which now allows large-scale epidemiological studies of this human disease. The first analysis of 57 infected patients revealed a high prevalence of a novel species – tentatively called – *”B. hominis”* and a large proportion of double infections.

We designed a Brachyspira species-specific PCR targeting the 16S rDNA that can identify all Brachyspira species that have been associated with infection in humans. Pettersson and colleagues identified three groups of spirochaetes that were closely related to, but based on 16S rDNA, phylogenetically different from, *Brachyspira aalborgi*. [16] These findings have been confirmed, but no epidemiological data exist. [14], [15], [17] Next to the 16S rDNA, the NADH oxidase gene (NOX gene) is frequently used to identify Brachyspira species. [41], [42] Mikosza et al. investigated the novel *“B. hominis”* sequences from both veterinary and human sources and sequenced the NOX gene. They found a topology very similar to Pettersson et al. in both the 16S rDNA sequences and the NOX gene. [17] Our results are in agreement with the results obtained by both Pettersson et al. and Mikosza et al.

Several authors have remarked that the 16S rDNA genes of the various Brachyspira species are highly similar, and that species differentiation based solely on 16S is difficult. [17], [43], [44] Therefore, finding a 16S sequence that differs from known species is a strong indicator that this in fact is a novel Brachyspira species. It is for this reason that the NOX gene is frequently used to identify the species of Brachyspiraceae. The described primers by Mikosza et al. target *B. aalborgi* and *“B. hominis”* sequences only, not amplifying any other Brachyspira species, therefore limiting direct species differentiation in human material, since *B. pilosicoli* also occurs in humans. Further studies are needed to better classify these novel sequences.

In 65 samples of 57 patients with histologically documented Brachyspira-infection we identified 17 (26.2%) samples with this novel 16S rDNA sequence of *“B. hominis”*, suggesting that more than the two hitherto described Brachyspira species are involved in human intestinal spirochaetosis. Moreover, these novel sequences were frequently (10/17; 58.8%) present in double- or even triple-infections, which may explain why they have not frequently been identified before. Our finding that this species was present as a mono-infection in seven patients and as a co-infection with other Brachyspira species in ten patients strongly suggest that this novel Brachyspira species is capable of infecting humans. However, more studies are needed to confirm these preliminary findings.

Ribosomal DNA sequencing is considered the gold standard in species determination. Our Bspp PCR provides rapid information with 100% accuracy for the presence (or absence) of Brachyspira species, and with 87.7% accuracy (95% confidence interval 0.76–0.94) for species identification. However, there were eight (out of 65; 12.3%) discrepancies between the predicted species based on the melting-curve and the sequence result. The melting-curves rely on a ∼1°C difference between species (figure 2; ∼79°C for *B. pilosicoli*, ∼80°C for *“B. hominis”* and ∼81°C for *B. aalborgi*). There are several known issues with SYBR-green. The T_m_ is influenced by a variety of factors. [45] Impurities in the extracted DNA, and/or higher concentrations of DNA might result in a minute shift of the melting temperature: there were never any discrepancies between the individual duplicates of samples. It is further supported by the observation that the melting-curve never shifted to the left, which would indicate a lower melting-temperature, but always to the right, indicating a higher melting-temperature (table 2). This is a known issue of SYBR-green based melting-curves. [45].

We acknowledge that the only true way of definitively calling a new species is to culture the bacterium. This is indeed a limitation that we are currently unable to solve, as we could not culture Brachyspira species from the formalin-fixed samples that were retrospectively identified in this study.

Based on the three Brachyspira positive faecal samples that were present in this study that could be correlated to positive biopsy samples, we conclude that this PCR allows for the testing of faeces. As this observation is based on concomitant material (biopsy and faeces) of three patients only, further studies are needed.

Although the analysis of 49 patients with Brachyspira infections suggested no correlation between species and physical complaints, the high prevalence of double infections, the small sample size of a the biased population (only patients of whom biopsies were taken for analyses of gastro-intestinal complaints) precludes strong conclusions on the clinical spectrum of disease caused by Brachyspira species. However, in pigs and poultry, *B. pilosicoli* is a known pathogen and the fact that the presence of *B. pilosicoli* was significantly associated with inflammatory changes in the colon-biopsy might suggest an immunologic reaction of the host against *B. pilosicoli*. Since only 49 patients had complete clinical records, no definitive pathogenic potential in humans can be attributed to *B. pilosicoli*. Further research into this finding is necessary.

In conclusion, we designed a real-time PCR for the immediate differentiation of Brachyspira species in human sample material, allowing for epidemiological studies in formalin-fixed paraffin-embedded material and faeces as well as routine, non-invasive clinical follow-up. Using this PCR, we investigated the epidemiology of the different Brachyspira species, and found a relatively high incidence of the novel *“B. hominis”* and a large proportion of double infections.

## Supporting Information

Figure S1 **Histopathological image of human intestinal spirochaetosis.** The spirochaetes are present as a ‘false brush border’ on the mucosal surface of the entire colon, as indicated by the arrow (haematoxylin and eosin stain, original magnification 630 times, bar equals 20 µm).
(TIF)

Table S1 **GenBank Accession Numbers**
(DOC)
