# Supplementary figures and images for: Development of a Real-Time PCR for Identification of Brachyspira Species in Human Colonic Biopsies
Source: PLoS One. 2012 Dec 20;7(12):e52281. doi: 10.1371/journal.pone.0052281 (PMC3527525; doi:10.1371/journal.pone.0052281)

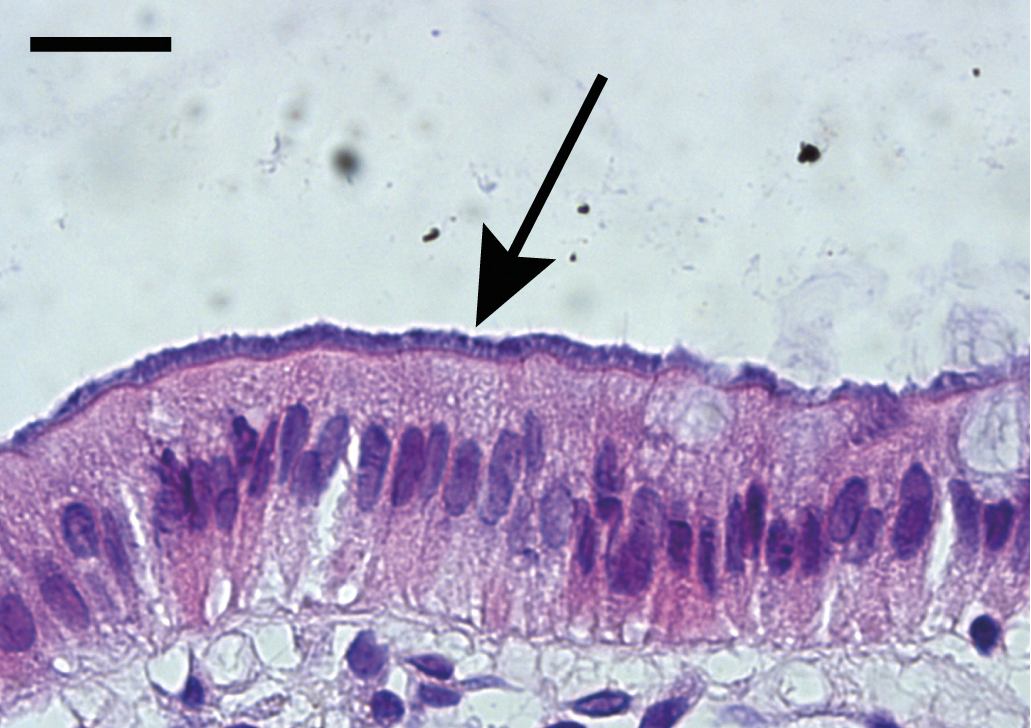

Supplement: Figure S1 — Histopathological image of human intestinal spirochaetosis. The spirochaetes are present as a ‘false brush border’ on the mucosal surface of the entire colon, as indicated by the arrow (haematoxylin and eosin stain, original magnification 630 times, bar equals 20 µm). (TIF) [file pone.0052281.s001.tif]
