# Supplementary material for: Development of a Real-Time PCR for Identification of Brachyspira Species in Human Colonic Biopsies
Source: PLoS One. 2012 Dec 20;7(12):e52281. doi: 10.1371/journal.pone.0052281 (PMC3527525; doi:10.1371/journal.pone.0052281)
Supplement: Table S1 — GenBank Accession Numbers (DOC) [file pone.0052281.s002.doc]

| ***Supplementary Table S1:*** *GenBank Accession Numbers* | |
| --- | --- |
| **Patient** | **GenBank Accession Number** |
| Patient_1 | JX446497 |
| Patient_2 | JX446498 |
| Patient_3 | JX446499 |
| Patient_4 | JX446500 |
| Patient_5 | JX446501 |
| Patient_6I | JX446502 |
| Patient_6III | JX446503 |
| Patient_7Ia | JX446504 |
| Patient_7Ib | JX446505 |
| Patient_7II | JX446506 |
| Patient_7III | JX446507 |
| Patient_8 | JX446508 |
| Patient_9I | JX446509 |
| Patient_9II | JX446510 |
| Patient_10 | JX446511 |
| Patient_11 | JX446512 |
| Patient_12 | JX446513 |
| Patient_13 | JX446514 |
| Patient_14 | JX446515 |
| Patient_15a | JX446516 |
| Patient_15b | JX446517 |
| Patient_16 | JX446518 |
| Patient_17a | JX446519 |
| Patient_17b | JX446520 |
| Patient_18 | JX446521 |
| Patient_19a | JX446522 |
| Patient_19b | JX446523 |
| Patient_20 | JX446524 |
| Patient_21 | JX446525 |
| Patient_22 | JX446526 |
| Patient_23 | JX446527 |
| Patient_24 | JX446528 |
| Patient_25 | JX446529 |
| Patient_26a | JX446530 |
| Patient_26b | JX446531 |
| Patient_27 | JX446532 |
| Patient_28 | JX446533 |
| Patient_29 | JX446534 |
| Patient_30I | JX446535 |
| Patient_30II | JX446536 |
| Patient_31 | JX446537 |
| Patient_32 | JX446538 |
| Patient_33 | JX446539 |
| Patient_34 | JX446540 |
| Patient_35Ia | JX446541 |
| Patient_35Ib | JX446542 |
| Patient_35IIa | JX446543 |
| Patient_35IIb | JX446544 |
| Patient_36Ia | JX446545 |
| Patient_36Ib | JX446546 |
| Patient_36IIa | JX446547 |
| Patient_36IIb | JX446548 |
| Patient_36IIc | JX446549 |
| Patient_37 | JX446550 |
| Patient_38 | JX446551 |
| Patient_39 | JX446552 |
| Patient_40 | JX446553 |
| Patient_41a | JX446554 |
| Patient_41b | JX446555 |
| Patient_41c | JX446556 |
| Patient_42 | JX446557 |
| Patient_43 | JX446558 |
| Patient_44 | JX446559 |
| Patient_45 | JX446560 |
| Patient_46 | JX446561 |
| Patient_47 | JX446562 |
| Patient_48 | JX446563 |
| Patient_49 | JX446564 |
| Patient_50 | JX446565 |
| Patient_51 | JX446566 |
| Patient_52 | JX446567 |
| Patient_53 | JX446568 |
| Patient_54 | JX446569 |
| Patient_55 | JX446570 |
| Patient_56 | JX446571 |
